# Uterine and Corpus Luteum Blood Flow Evaluation Prior to Uterine Flushing in Llama Embryo Donors

**DOI:** 10.3389/fvets.2020.597960

**Published:** 2020-11-17

**Authors:** Enzo G. Zampini, María F. Gallelli, María G. Chaves, Deborah M. Neild, Mariana Gambarotta, Marcelo H. Miragaya, Virginia L. Trasorras

**Affiliations:** ^1^Universidad de Buenos Aires (UBA), Facultad de Ciencias Veterinarias (FCV), Instituto de Investigación y Tecnología en Reproducción Animal, Cátedra de Teriogenología, Buenos Aires, Argentina; ^2^Consejo Nacional de Investigaciones Científicas y Técnicas, Buenos Aires, Argentina; ^3^Universidad de Buenos Aires (UBA), Facultad de Ciencias Veterinarias (FCV), Cátedra de Bioestadística, Buenos Aires, Argentina

**Keywords:** camelids, embryo transfer, Doppler ultrasonography, pregnancy, biotechnologies

## Abstract

The aim of this study was to assess the uterine blood flow (UBF) and corpus luteum blood flow (CLBF) in llamas 8 days post-mating, using color-Doppler ultrasonography (CDU), to determine the possible relationship between vascularization and the presence of an embryo. Adult females (*n* = 25) were used to monitor ovarian dynamics by palpation and transrectal ultrasonography until detection of a ≥6 mm growing follicle. Females were randomly assigned to one of two groups: Group I (*n* = 19), were mated and ovulation was induced by a single dose of buserelin (GnRH analog) that same day (Day 0); and Group II (*n* = 6), only ovulation was induced (control). On Day 8, UBF and CLBF were evaluated transrectally in both groups. The color-flow images obtained were analyzed with Image J1.52a software to determine the vascularization area and the percentage of corpus luteum with blood flow emission (CLBF%) together with the percentage for each uterine horn (UBF%). Statistical analysis was performed using an ANOVA test. In Group I, uterine flushing was performed to obtain the embryos, thus dividing the females into Group I+ (*n* = 10), when an embryo was recovered and Group I- (*n* = 9), when no embryo was recovered. Embryo recovery rate was 52.63% (10/19). In Group I+, UBF% was significantly higher compared to Group I- and Group II (*P* <0.05). UBF appears to be a good predictor for embryo presence, with an area under the curve (AUC) of 0.9 and an optimal cut-off value of 9.37% (with a sensitivity of 90% and specificity of 88.9%). The CLBF% did not differ between groups (*P* > 0.05). In conclusion, it is possible to detect a local increase of UBF in the presence of an embryo on day 8 post-mating in llamas. This could be useful to achieve an early pregnancy diagnosis or to decide whether to carry out the uterine flushing in a llama embryo transfer program.

## Introduction

Llamas are monotocous and have a prolonged gestation period [335–360 days; (1)], hence early pregnancy diagnosis would allow a greater efficiency to obtain one offspring per female per year when conception fails and gives better productive indices. To date, the methods used to diagnose gestation in South American camelids (SAC) are limited. One of the methods that is widespread among SAC producers is to observe the sexual behavior of the females when confronted with a male, from 11 to 13 days after natural mating (2, 3). Although this is an inexpensive technique as it doesn't require sophisticated tools or equipment, behavior interpretation is subjective and prone to error. There are even dominant females that, in absence of high progesterone levels, still reject the male without being pregnant. In addition, this method does not provide information on the number of embryos or fetuses present, their state of development or health. Another diagnostic tool is measuring plasma progesterone levels 11–13 days post-mating (4, 5) as it reflects the presence of a functional corpus luteum (CL), necessary to maintain pregnancy in SAC (6). However, validated progesterone dosage tests for SAC are scarce and difficult to access and the time lag between blood collection and availability of the results is also problematical. Furthermore, reproductive disorders that prolong the luteal phase, such as spontaneous ovulations and luteinized cystic follicles, can produce a 15% of false positive results (5). But, similarly to the first method, this technique doesn't provide information on the number, development, and health of the embryo or fetus either. Pregnancy evaluation by transrectal palpation is possible 35 days after mating in llamas, with greater accuracy 45–50 days after mating (2, 3, 7). This is a simple, low-cost maneuver that although provides fetal viability data, the estimation of gestational age, and therefore prediction of probable date of parturition, are not precise (8). So far, the most effective, precise and early method for diagnosing gestation in SAC is transrectal ultrasonography in Brightness mode (B-mode), which allows visualization of the embryo vesicle 12–14 days after mating, although is more precise as of 16–23 days (9). Unlike the above methods, it not only gives information regarding the number, development and viability of the embryo/s or fetus/es, it also allows one to approximate gestational age (10), even when the date of natural mating is unknown, and hence estimate the probable date of parturition.

In SAC, the embryo reaches the uterine lumen as a hatched blastocyst, between 6 and 6.5 days after ovulation (11, 12), and produces increasing quantities of estradiol-17β during days 7–15 of gestation (13). According to Ford (14), the estradiol produced by embryos of domestic species is responsible for the local increase in uterine blood flow (UBF) registered during early pregnancy. However, it is not known if there is a connection between the presence of the embryo and uterine blood flow in llamas in the early stages of gestation. Color Doppler ultrasonography (CDU) not only allows evaluation of the bidimensional structure of the different organs but also offers the possibility of observing their vascular system (15) as it overlays the color signals of blood flow over the B-mode images (16). To calculate the degree of vascularization of an organ, the percentage of tissue with color signals can be estimated or the images that are captured can be processed and analyzed by a computer using software that allows an objective calculation of the number of colored pixels over the B-mode area (17). The aim of this study was to assess the uterine blood flow and corpus luteum blood flow in llamas, 8 days post-mating using color Doppler ultrasonography to determine the possible relationship between vascularization and the presence of an embryo.

## Materials and Methods

### Animals

Non-pregnant, non-lactating female llamas (*n* = 25) ranging between 4 and 12 years of age and with an average body weight of 120 ± 22 kg were used in this study. All animals were in a good nutritional status (body condition), both healthy and reproductively active at the time of the trial. Females were kept separate from the males and fed with hay and water *ad-libitum*. The study was conducted between March 2018 and December 2019 at the Faculty of Veterinary Sciences of the University of Buenos Aires, Buenos Aires, Argentina, situated 34° 36' S and 58° 26′ W at sea level. This study was approved by the Committee for the Use and Care of Laboratory Animals (CICUAL, 2017/67) of the Faculty of Veterinary Sciences of the University of Buenos Aires.

### Experimental Design

Ovarian dynamics were monitored by transrectal palpation and ultrasonography (Berger LC 2010 plus with a 5 MHz linear-array electronic transducer) until the presence of a growing follicle ≥6 mm was detected. At that moment, all females received a single IV dose of 8 μg of buserelin (GnRH analog; Receptal®, Intervet, Buenos Aires, Argentina) (day 0) to induce endogenous LH release and ovulation. After buserelin injection, llamas were randomly divided in two groups: Group I (*n* = 19), embryo donor females were mated with a male with proven fertility and uterine flushings were done for embryo recovery on day 8; Group II (*n* = 6), females without natural mating (control group). Ovulation was confirmed using transrectal ultrasonography based on the disappearance of the dominant follicle on day 2, and control of the ovarian dynamics was carried out every other day (B-mode ultrasonography) until day 8.

### Uterine and Corpus Luteum Vascularization

All females were examined by transrectal CDU (MyLab^TM^ 30Gold VET ESAOTE, attached to a 5 MHz linear-array electronic transducer) in order to assess the UBF and corpus luteum blood flow (CLBF) on day 8. The settings (B-mode frequency: 5 MHz with a depth of 8 cm and a gain of 52%; CFM pulse repetition frequency: 1.4 KHz and a gain of 70%) were standardized and remained constant for all examinations. Briefly, the transducer was placed over the middle segment of each uterine horn (UH) as described in llamas (18, 19), to display signals for blood flow in the vessels of all the endometrium, myometrium, and perimetrium, and over the CL. A 3 s video-clip of the vascularization of each structure was registered and downloaded. The video-clips were examined frame by frame (Adobe Premiere Pro CS6®) to select images that showed the maximum vascular signal, three of each UH and three over the maximum cross-sectional area of the CL. A total of 225 images (nine images per female) were saved in tagged image file format (TIFF) and analyzed by an operator without knowledge of the identity of each animal or the result of uterine flushing, using ImageJ 1.52i software (National Institute of Health, USA). The degree of vascularization was estimated by measuring the colored area (cm^2^) of the vascular flow signals (Doppler mode) over the left UH, right UH and the CL area (cm^2^) (B-mode). Thus, percent area of vascularization was calculated by the following equation: percent of blood flow area (BF%) = (vascular area/total organ's area) ×100. The average of the three images was considered as the final value for each uterine horn (UBF%) and for the CL (CLBF%) of each animal. In females with two CLs, we calculated the CLBF% of both and considered the average. Uterine flushing was performed in Group I after Doppler ultrasonographic evaluation.

### Embryo Recovery and Evaluation

In Group I, uterine flushing was carried out non-surgically for embryo recovery, 8 days after mating (20). The maneuvers were performed with the female either standing or in sternal recumbency. The animal was restrained in stocks, the tail was wrapped and the rectum was emptied of feces. The perineum was then scrubbed using a hypoallergenic detergent, rinsed carefully with clean water and then dried. Restless females received 0.2 mg/kg IV xylazine (Xilazina® 10%, PRO-SER S.A., Buenos Aires, Argentina) before flushing. A Foley catheter (Vortex^TM^; Agtech, Inc.; Manhattan, USA.) 12 or 16 Fr, according to female size, with a stylet inserted into the catheter to keep it from bending during recto-vaginal manipulation, was used. Uterine flushing was done by placing the catheter cuff cranial to the internal cervical os and insufflating it with 5 or 10 ml of air (depending on catheter gauge). The whole uterus was flushed 4 to 5 times using Ringer-Lactate solution (Laboratorios Rivero, Buenos Aires, Argentina), previously warmed (30–35°C), with a total volume of 500 ml. The recovered medium was filtered through a 70 μm EmCon^TM^ filter (Agtech, Inc.) for embryos. The fluid in the flushing filter was placed in warmed reticulated Petri dishes and embryos were identified using a stereomicroscope. Embryos were measured and classified according to their morphology following the criteria set by Tibary and Anouassi (21), using a grade scale from 1 to 5. After flushing, donor females received 250 μg IM of PGF_2α_ (cloprostenol; Ciclase DL®, Syntex S.A., Buenos Aires, Argentina) to induce luteolysis. The number of embryos collected, and positive pregnancy diagnoses were recorded. Thus, females were divided into Group I+, when an embryo was recovered (positive uterine flushing) and Group I-, when no embryo was recovered (negative uterine flushing).

### Statistical Analysis

Statistical analyses were performed using InfoStat software (version 2020; FCA, National University of Córdoba, Argentina). After verifying normal distribution of the data using a Shapiro-Wilks test, an ANOVA test was applied to evaluate uterine and CL vascularization. These data were subjected to Tukey's test to determine significance. In all females, the uterine horn with highest UBF% was chosen. Furthermore, we performed receiver operating characteristics (ROC) analyses focusing on UBF% on day 8, to identify the optimal cutoff value for predicting pregnancy. We evaluated every candidate cutoff value for the optimal cutoff value by geometric distance for 100% sensitivity and specificity. The optimal cutoff value was determined using the data point that minimized the distance (22). Prognostic value was expressed as the area under the curve (AUC) with a 95% confidence interval and a test of significance. A paired *T*-test was used to evaluate the relationship between the blood flow of each uterine horn and the location of the CL in the females in Group I+. Pearson or Spearman's correlation analysis were used to compare various parameters (Follicle and CL diameter, CLBF). The correlations between parameters were classified according to Taylor (23) as weak (*r* ≤ 0.35), moderate (*r* = 0.36–0.67) or strong (*r* = 0.68–1.00). Values were expressed as mean ± SEM. Differences were considered significant when *P* < 0.05.

## Results

### Embryo Recovery Rate and Quality

Embryo recovery rate was 52.63% (10/19), with a total of 11 embryos recovered from 19 flushing procedures (two embryos were obtained from one female). Hence, females in Group I were divided into Group I+: *n* = 10 and Group I-: *n* = 9. Of the 11 recovered embryos, nine were grade I, one was grade II and one was an arrested morula (grade V). Embryo size ranged from 350 to 1,500 μm.

### Uterine Blood Flow in Pregnant and Non-pregnant Females

**Figure 1:**
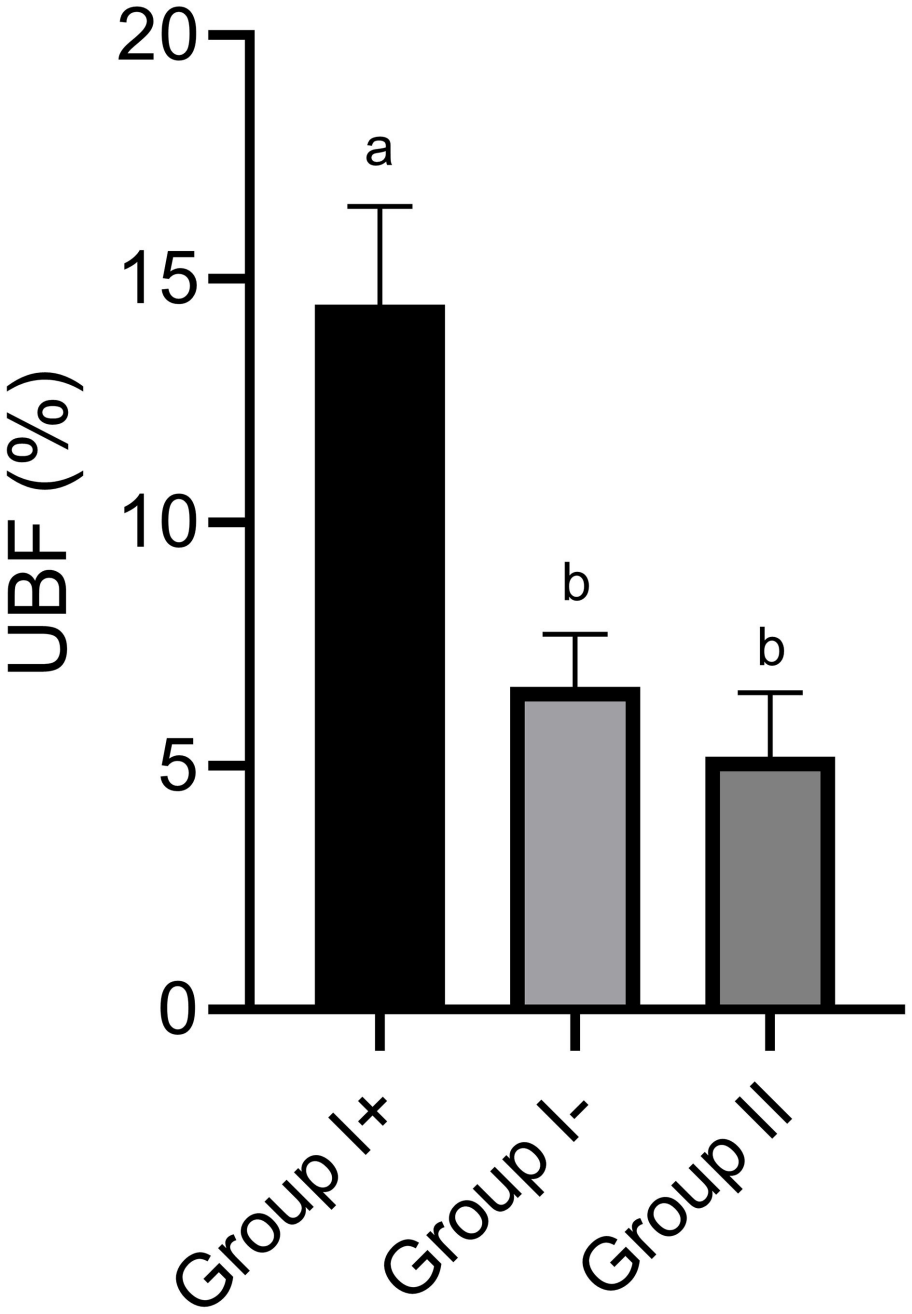
Uterine blood flow (UBF) in the three groups: llamas with an embryo recovered (Group I+), llamas without an embryo recovered (Group I-) and control group (Group II, ovulated, non-mated females). Values are mean ± SEM. ^a, b^Groups with different letters are significantly different (*P* < 0.05).

**Figure 2:**
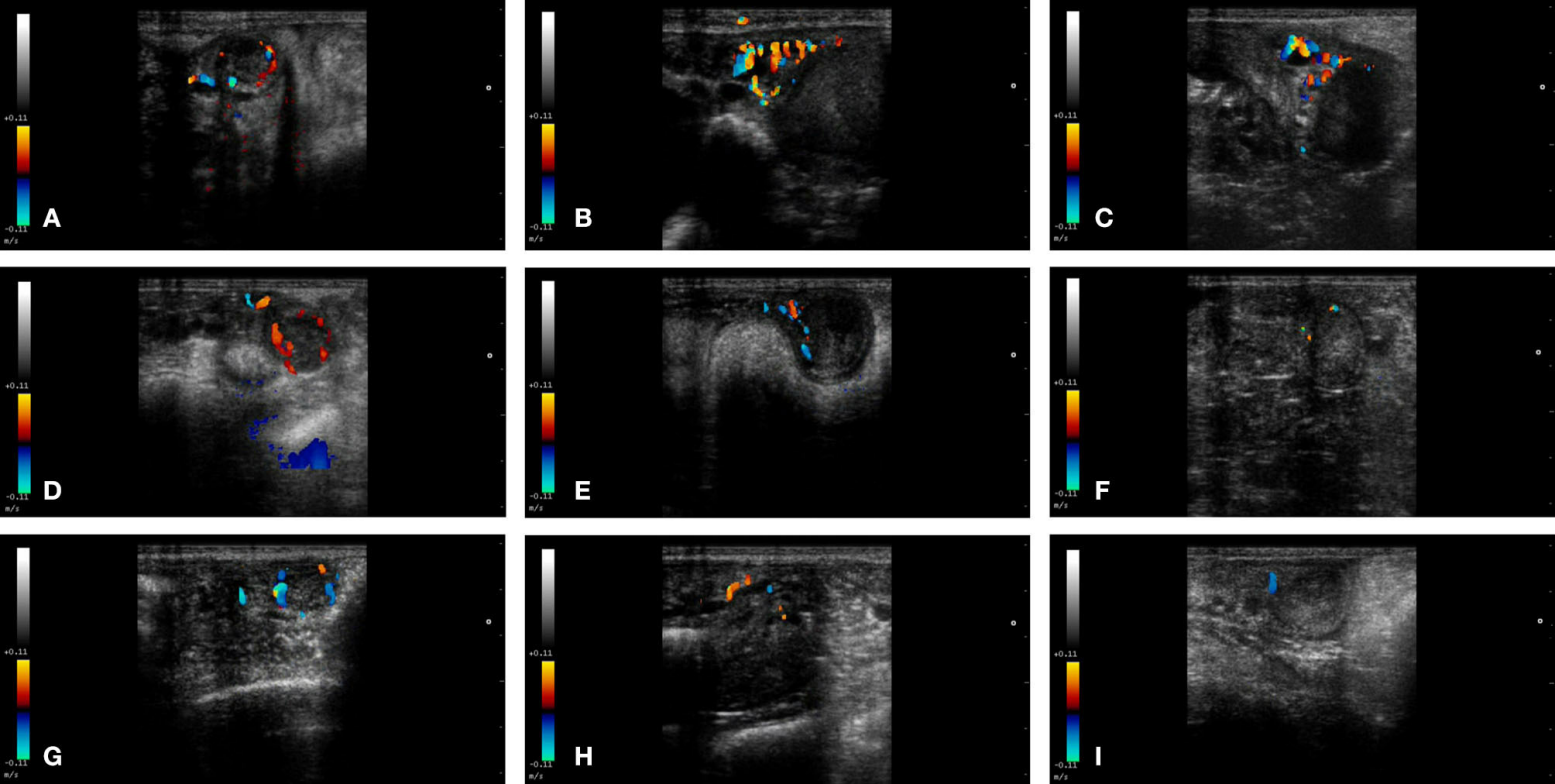
Color-Doppler images from a female llama from each group in the study. From left to right: CL, left uterine horn and right uterine horn. Group I+ **(A–C)**, Group I- **(D–F)**, and Group II **(G–I)**.

In Group I+, UBF% was significantly higher (14.5 ± 1.5%) than in Group I- (6.6 ± 1.2 %) and Group II (5.19 ± 1.94 %; *P* < 0.05). Moreover, no differences were found between Group I- and Group II (*P* > 0.05) (Figures 1, 2).

### Predicting the Presence of an Embryo Using a ROC Curve

**Figure 3:**
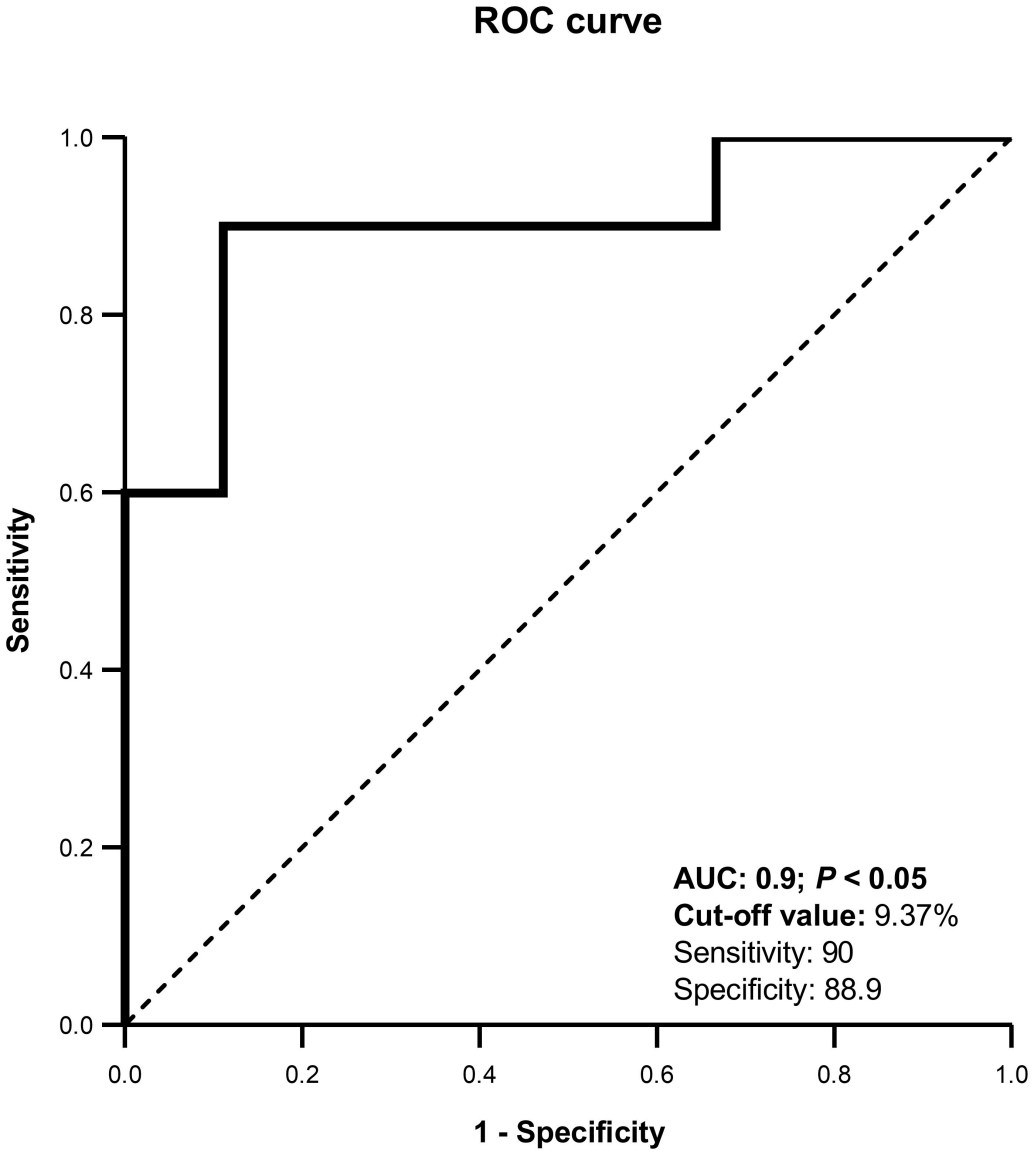
Receiver Operating Characteristic (ROC) curve for uterine blood flow (%). AUC, Area under the curve. The curve was able to detect pregnant llamas when UBF% was >9.37%, with a sensitivity of 90% and a specificity of 88.9%.

To prepare the Receiver Operating Characteristic (ROC) curve, the uterine horn with highest UBF% on day 8 from each female was taken. Differentiation between pregnant and non-pregnant llamas was possible with this technique with an area under the curve (AUC) of 0.900. The optimal cut-off value was 9.37%, with a sensitivity of 90% and specificity of 88.9% (Figure 3).

### Blood Flow of Each Uterine Horn and Its Relationship With the Location of the CL in Pregnant Females

Females in Group I+ showed no significant differences in UBF% between both uterine horns irrespective of the location of the CL (*P* > 0.05). However, those with a CL in the left ovary showed a tendency to have a higher UBF% in the left UH compared to the right UH (*P* = 0.06), whereas when the CL was in the right ovary, UBF% was similar between uterine horns (*P* = 0.97).

### Corpus Luteum Diameter and Vascularization in Pregnant and Non-pregnant Females

**Figure 4:**
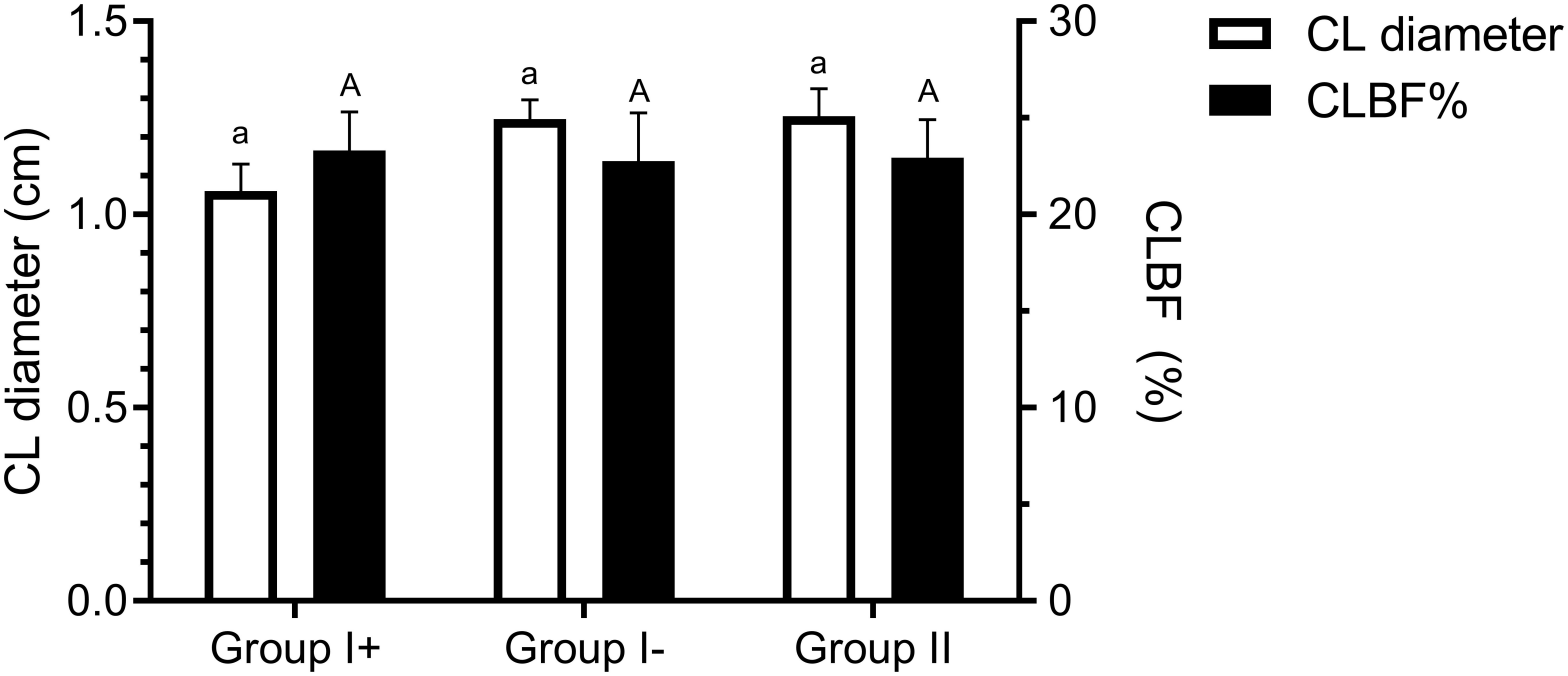
Corpus luteum diameter (cm) and blood flow (CLBF%) on day 8 in the three groups: llamas with an embryo recovered (Group I +), llamas without an embryo recovered (Group I-) and control group (Group II, ovulated, non-mated females). Both variables were expressed as mean ± SEM. ^a, A^indicate no significant differences between groups for both parameters (*P* > 0.05).

Diameter of the CL on day 8 was similar between all groups (Group I+: 1.06 ± 0.07 cm; Group I-: 1.25 ± 0.06 cm; Group II: 1.25 ± 0.07 cm) (*P* = 0.76). Neither were significant differences detected in CL blood flow (CLBF%) between Groups I+ (23.29 ± 2.05 %), I- (22.76 ± 2.16 %) and II (22.93 ± 2.64 %) (*P* = 0.98) (Figure 4).

### Correlation Analysis

Follicular diameter on day 0 was moderately correlated to CL diameter on day 8 (Spearman's coefficient of correlation: sρ = 0.61; *P* = 0.0013) but was not correlated to CLBF% on day 8 (Pearson's coefficient of correlation: ρ = −0.28; *P* = 0.18); neither was CL diameter on day 8 correlated to CLBF% on the same day (ρ = 0.08; *P* = 0.72).

## Discussion

Embryo transfer is a powerful tool for increasing the number of offspring from a specific donor, but it is also becoming increasingly important for the genetic improvement of livestock herds (20, 24–26). Flushing for embryo recovery from the donor llama's uterus is normally performed on day 8 post-mating, without a previous pregnancy diagnosis. Using CDU, we observed that mated llamas with an embryo recovered (Group I+), had a significantly higher UBF% than mated females without an embryo recovered (Group I-) and llamas that ovulated but not were mated (Group II; control). This increase in UBF in pregnant females vs. non-pregnant females was also observed in embryo donor mares, where Doppler ultrasonography was used to make an early pregnancy diagnosis 8 days after artificial insemination, prior to embryo recovery. They established a cut-off value of 35.55 mm^2^ to distinguish adult pregnant mares from those that were not gestating, with a 97.2% sensitivity and an 85.7% specificity (27). In inseminated buffalo, increases in UBF were registered after ovulation, with a marked difference observed between pregnant and non-pregnant females from 7 days post-ovulation; hence it was possible to diagnose pregnancy on that date (28). An adequate uterine perfusion is essential in mammals for a correct development of gestation (29), as according to Habara et al. (30), uterine blood supply could regulate endometrial receptivity. It has been suggested that quantifying the increase in uterine blood flow during the luteal phase could be a good predictor for the success of embryo implantation (31). Silva et al. (17) observed that the UBF in mares increased locally at the site of the embryo during the migration phase (days 6 to 16 post-ovulation). They attributed this increase to the possible vasodilator action exerted by vasoactive agents, such as estrogens and prostaglandins, secreted by the equine embryo during that period. This increase in vascularization of the uterine horn ipsilateral to the embryo was also observed by Honnens et al. (32) in pregnant dairy cows from day 11 of gestation. Although the llama with a grade V embryo recovered (arrested morula) was classified in to Group I+ (positive uterine flushing), its UBF was 5.4%, comparable to the mean value registered in mated females without an embryo recovered (Group I-: 6.6%) and llamas that ovulated but not were mated (Group II: 5.19%). This could be due to the metabolic inactivity of the arrested morula and thus, to the lack of secretion and consequent action of its vasoactive agents.

In SAC, ovulations occur with equal frequency from both ovaries, however, most pregnancies are located in the left uterine horn [alpaca: 97.5 and 99.3% with a CL in the right and left ovaries, respectively, (33, 34); llama: 100%, (6)]. For this reason, an embryo resulting from an ovulation in the right ovary needs to migrate or emit some type of signal from the right uterine horn to the left to somehow indicate its presence to both uterine horns and thus prevent luteolysis (11, 20, 35, 36). Although the antiluteolytic signal has not yet been identified, it is believed that the estradiol-17β secreted by the blastocyst could be involved (37). The possible migration and resulting contact with both uterine horns of the embryo proceeding from an ovulation in the right ovary could explain the increase in blood flow registered in both uterine horns in the females of Group I+ with a CL in the right ovary. Powell et al. (13), suggested that llama embryo mobility through the uterus could be mediated by the estradiol-17β it secretes during the early stages of pregnancy, generating a localized increase in myometrial contractility and so allowing its propulsion toward the left uterine horn. Embryo estrogens would exercise this action by binding to the estrogen receptor β, whose expression in the myometrium and perimetrium is increased in the presence of a CL and is greater in pregnant vs. non-pregnant females (35). Whereas, if ovulation occurs in the left ovary, the embryo would not need to migrate because the right uterine horn is incapable of lysing a CL in the left ovary (11, 20, 36). Estradiol has a potent vasodilator effect (18, 38, 39) and it is possible that for this reason, the females in Group I+ with a CL in the left ovary register higher levels of blood flow in the ipsilateral uterine horn. However, increasing the number of animals evaluated could take this difference in UBF% between uterine horns to become statistically significant.

The positive correlation between the follicle diameter on day 0 and the CL diameter on day 8 that we detected was also observed in cows (40, 41), ewes (42), and mares (43–45) and could be because the size of the pre-ovulatory follicle would regulate the size of the CL in its early stages, mainly due to a spatial constraint (46). Nevertheless, the vertical diameter of the CL on day 8 was similar between pregnant females (Group I+) and non-pregnant females (Groups I- and II). According to previous studies in llamas (47, 48), after mating or inducing ovulation, the CL that is formed reaches its maximum diameter 10 days after the stimulus, both in pregnant and non-pregnant females. Only after this date does the CL commence to decrease in size in non-pregnant females while maintaining a plateau in pregnant animals and the difference between both groups becomes evident only after 14 days. Similarly, CLBF% on day 8 showed no significant differences between pregnant and non-pregnant females. Considering that the CL is the most highly vascularized temporary tissue of the body and receives the greatest rate of blood flow per unit of tissue (49), no apparent differences between pregnant and non-pregnant females in luteal blood flow would be expected during early angiogenesis in CL development (40). Our findings coincide with a recent report by Gallelli et al. (48), who evaluated CL blood flow using CDU in llamas after natural mating and observed that the CLBF was similar in all females up to 8 days post-mating. After that, CLBF remained high in pregnant females, while in non-pregnant animals it decreased dramatically until it disappeared between 14 and 16 days after mating. The difference in CLBF between pregnant and non-pregnant llamas was only evident after day 12 (48). Similar CLBF profiles between non-pregnant females were also observed using Doppler ultrasound in llamas (50, 51), alpacas (52), and dromedaries (53, 54), after inducing ovulation. In these cases, CLBF reached maximum values between 7 and 9 days after induction of ovulation and after that, they began to descend and reached basal levels between days 12 and 14. In all cases, CLBF reduction in non-pregnant females coincided with the process of luteolysis, which occurs over this period in these species. According to different authors, CL function (progesterone production) would be determined by its vascularity more than by its size [llamas: (48); dromedaries: (53); bovines: (55–57)]. In cows, de Tarso et al. (40, 41) evaluated the diameter and blood flow of the CL and found a moderate correlation (ρ = 0.43 a 0.6) between both parameters. However, in our study no correlation between the diameter of the CL and its blood flow was observed on day 8, similar to what had been previously reported by Gallelli et al. (48).

According to our study, CDU would predict the outcome of transcervical uterine flushings based on the UBF% 8 days after natural mating in llamas. By constructing a ROC curvewith an AUC of 0.900 we established that, if 9.37% is used as the cut-off value, it is possible to estimate the result of a uterine flushing with a 90% sensitivity and an 88.9% specificity. This would provide valuable information when deciding whether or not to perform a uterine flushing in an embryo transfer program. In addition, females diagnosed as pregnant could be separated early to adopt management and nutrition maneuvers appropriate to this new reproductive status, first adapting their nutritional plan and minimizing stress situations in order to prevent embryo mortality (58) which is high in these species (3). On the other hand, non-pregnant females detected early could be separated to be mated again. However, due to the high percentage of early embryo losses registered in these species, it would be best to combine this early detection method with other methods implemented later on, such as B-mode ultrasonography or transrectal palpation, to confirm pregnancy diagnosis.

## Conclusion

In conclusion, the results of the present study indicate that evaluation of uterine blood flow by color Doppler ultrasound combined with computer assisted analysis of images are reliable techniques for detection of early pregnancy prior to embryo recovery on day 8 post-mating in llamas.

## Data Availability Statement

The raw data supporting the conclusions of this article will be made available by the authors, without undue reservation.

## Ethics Statement

The animal study was reviewed and approved by Committee for the Use and Care of Laboratory Animals (CICUAL).

## Author Contributions

VT and EZ contributed conception, design of the study, and wrote the manuscript. EZ, VT, and MFG provided help with field work. MG performed the statistical analysis. MC, DN, and MM made critical revisions to the paper. DN contributed to language editing. All authors contributed to the article and approved the submitted version.

## Conflict of Interest

The authors declare that the research was conducted in the absence of any commercial or financial relationships that could be construed as a potential conflict of interest.
